# Supplementary figures and images for: Opening the black box of bird-window collisions: passive video recordings in a residential backyard
Source: PeerJ. 2022 Dec 20;10:e14604. doi: 10.7717/peerj.14604 (PMC9784330; doi:10.7717/peerj.14604)

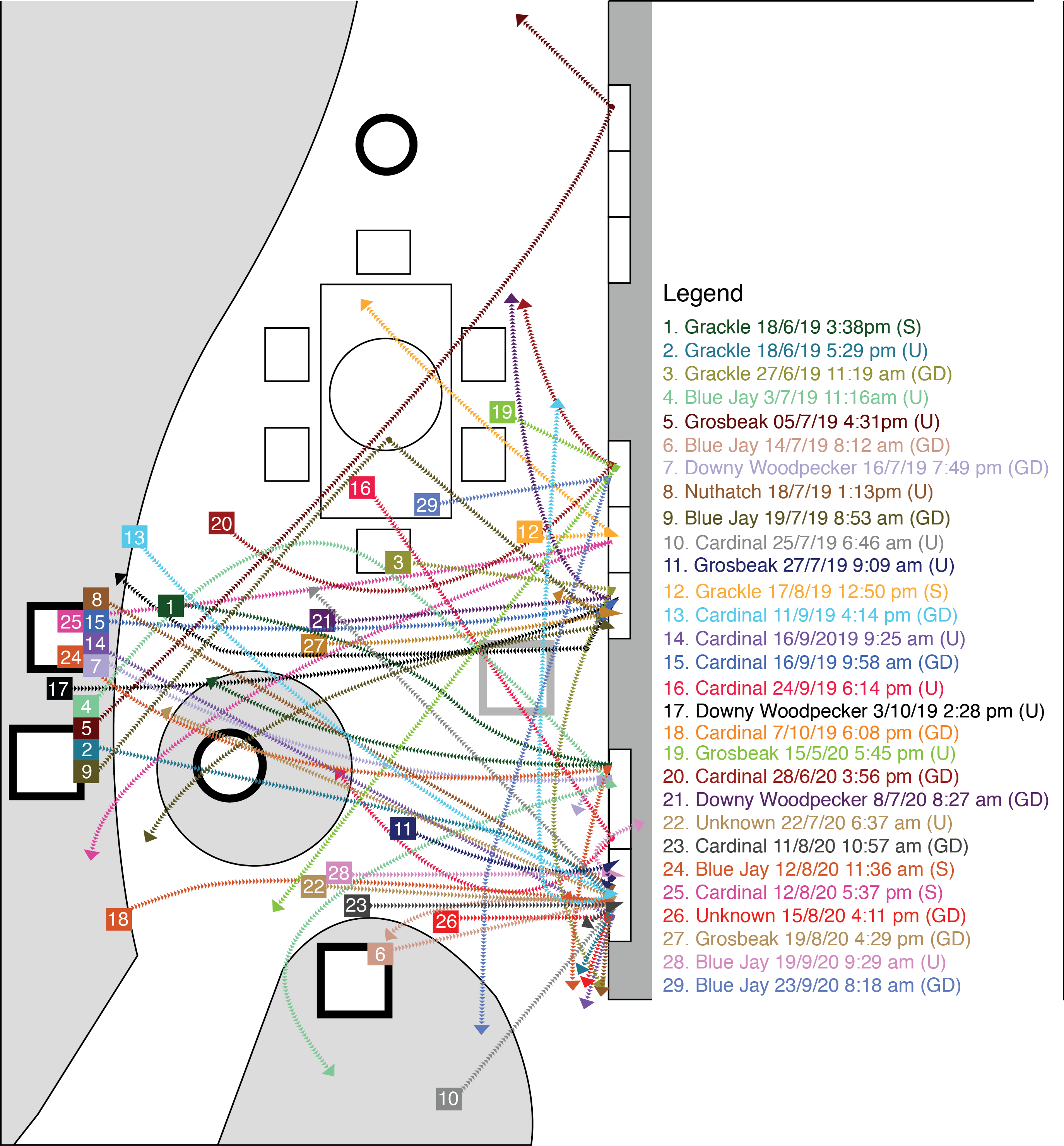

Supplement: Supplemental Information 1 — Colours are used to distinguish individual birds. Coloured boxes containing numbers in the figure indicate the starting position of the bird. Events in the legend are ordered chronologically by date. In the legend, (U) = bird collided with the upper transom windows above the door, (GD) = bird collided with the glass door, (S) = bird collided with the screen over a glass door. [file peerj-10-14604-s001.png]

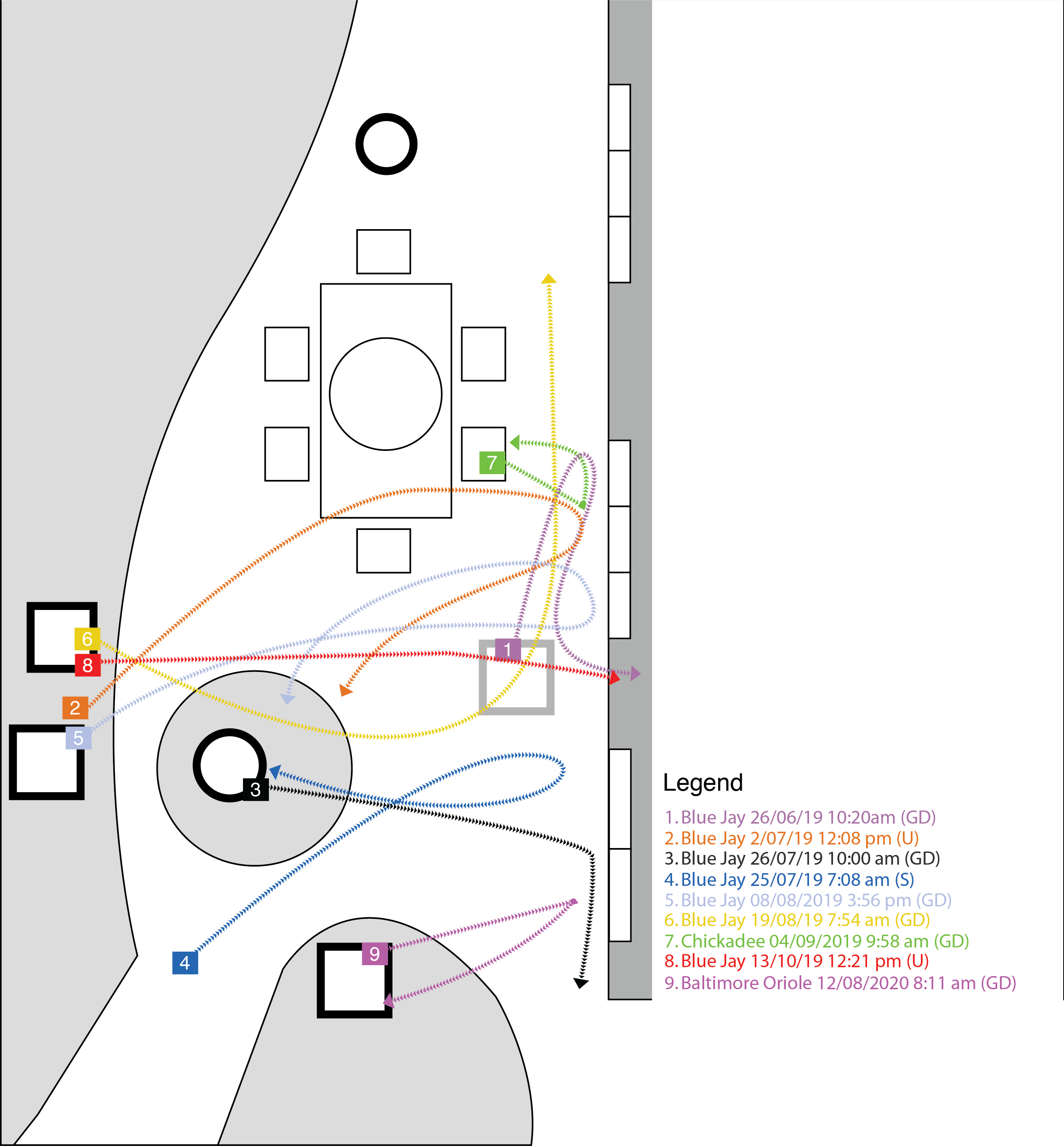

Supplement: Supplemental Information 2 — Colours are used to distinguish individual birds. Events are ordered chronologically by date. In the legend, (U) = bird approached the upper transom windows above the door, (GD) = bird approached the glass door, (S) = bird approached the screen over glass door. [file peerj-10-14604-s002.png]

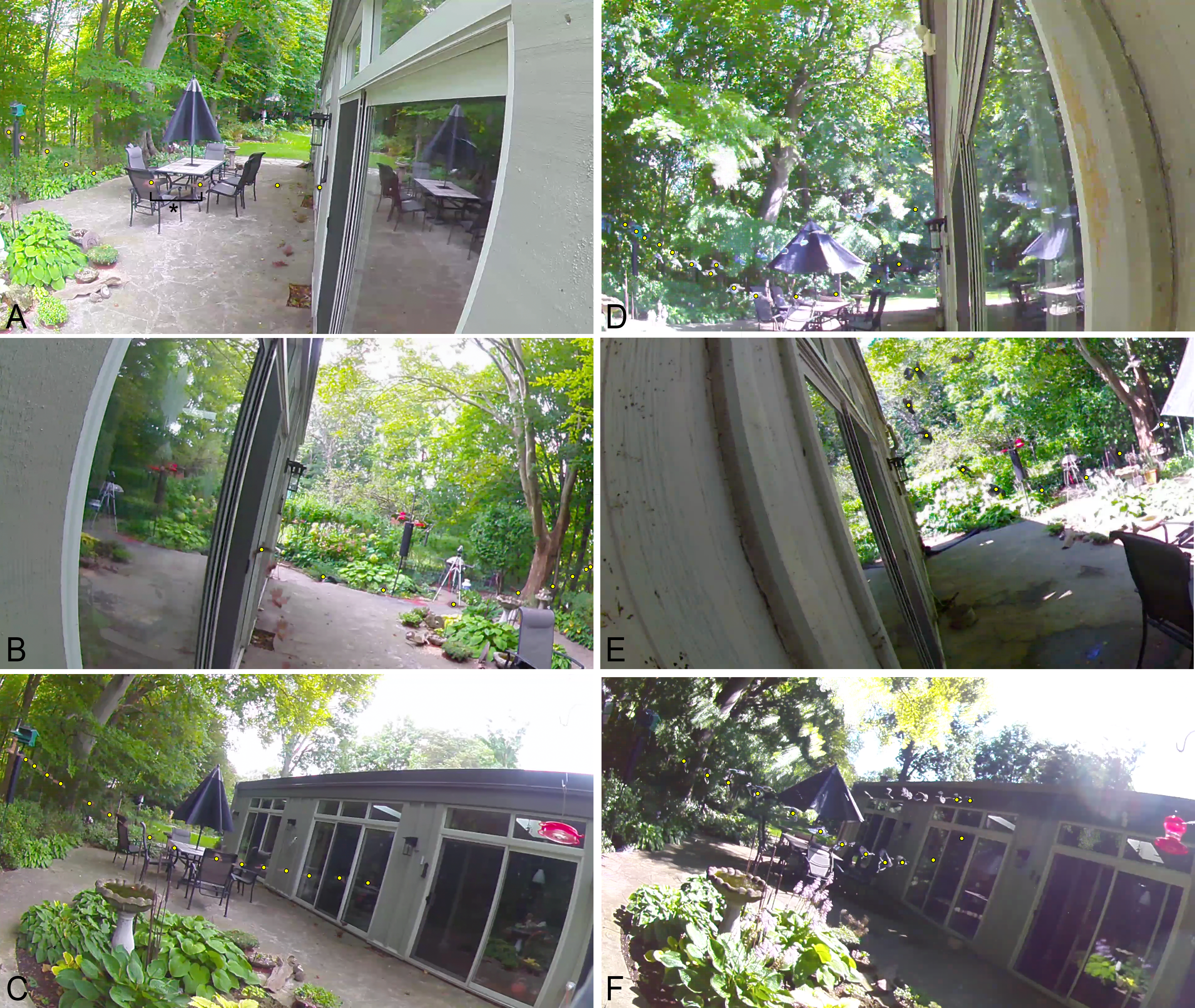

Supplement: Supplemental Information 3 — A, B and C show overlaid video frames from three cameras of the same collision event, a Northern Cardinal (#15 in Fig. S1). D, E, and F show overlayed video frames from three cameras of the same near miss event, a Blue Jay (#5 in Fig. S2). The position of the bird across frames is highlighted with yellow markers. For calculating velocity, measure the displacement of the bird between consecutive video frames (compare with measurements given in Fig. 1). Then, divide displacement by 0.042 s (duration between frames at 24 fps) to determine momentary velocity. Take the mean velocity across frames up to the point when the bird was nearest to the window. Note that images shown here are composites of video frames provided for illustration purposes; birds in motion are more clearly visible in video footage.[b] [file peerj-10-14604-s003.png]
