# Supplementary material for: Opening the black box of bird-window collisions: passive video recordings in a residential backyard
Source: PeerJ. 2022 Dec 20;10:e14604. doi: 10.7717/peerj.14604 (PMC9784330; doi:10.7717/peerj.14604)
Supplement: Supplemental Information 5 [file peerj-10-14604-s005.docx]

# required R packages:

# requires (lme4)

# requires (afex)

# requires (tidyverse)

# requires (psych)

# requires (optimix)

# requires (ggpubr)

# requires (broom.mixed)

setwd("C:/Rfiles") # sets folder

rm(list = ls ()) # clears R's memory

binarydata <- read.csv("peerj-74911-data.csv", na.strings = c("","NA"), header=TRUE) # opens file

set_sum_contrasts() # criterion for running the contrasts in afex

View(binarydata) # view dataset

str(binarydata) # summary of the types of variables and their values

# changes variables to factor vars

binarydata$species <- as.factor(binarydata$species)

# making dependent vars numerical factors

binarydata$collnear <- as.numeric(as.character(binarydata$collnear))

binarydata$collafter <- as.numeric(as.character(binarydata$collafter))

# absolute values for horizontal angle

binarydata <- mutate(binarydata, horizangleabs = abs(horizangle - 90))

#correlations between independent continuous factors in the dataset

ind.cont1 <- binarydata[c("horizangleabs", "time_dec", "velocity")]

corr.test(ind.cont1, use = "pairwise", method = "pearson", adjust = "none")

# no high correlation coefficients so we include them all

str(binarydata)

# assessing normality of independent continuous factors

qqnorm(binarydata$velocity)

qqline(binarydata$velocity)

qqnorm(binarydata$horizangleabs)

qqline(binarydata$horizangleabs)

qqnorm(binarydata$time_dec)

qqline(binarydata$time_dec)

# providing descriptive statistics on the independent factors

descr(binarydata$velocity, style="rmarkdown")

descr(binarydata$horizangleabs, style="rmarkdown")

descr(binarydata$time_dec, style="rmarkdown")

#Centering the continuous independent variable dist.from.media

MyNorm <- function(x){ (x-mean(x, na.rm = TRUE))/sd(x, na.rm = TRUE)}

#Add na.rm = TRUE to deal with datasets with NA's

binarydata$velocityc <- MyNorm(binarydata$velocity)

binarydata$horizangleabsc <- MyNorm(binarydata$horizangleabs)

binarydata$time_decc <- MyNorm(binarydata$time_dec)

###### Dependent variable: probability of collision with window when birds flew close to the window

# model with centered variables to obtain effect sizes (odds ratios)

collnearm1 <- mixed(collnear ~ velocityc + horizangleabsc + time_decc + (1|species), data = binarydata, method = "LRT", family = binomial, expand_re = TRUE)

anova(collnearm1)

# model has convergence issues

# we assess the potential convergence issues in the following lines

#### https://rstudio-pubs-static.s3.amazonaws.com/33653_57fc7b8e5d484c909b615d8633c01d51.html

# we check for singularity issues

tt <- getME(collnearm1$full_model,"theta")

ll <- getME(collnearm1$full_model,"lower")

min(tt[ll==0])

# singularity does not seem to be a problem because theta is much hight than zero

# we increase the number of iterations of the model

ss <- getME(collnearm1$full_model,c("theta","fixef"))

m2 <- update(collnearm1$full_model,start=ss,control=glmerControl(optCtrl=list(maxfun=2e4)))

# now there are no convergence issues

##### we try different optimizers following this approach:

## https://rstudio-pubs-static.s3.amazonaws.com/33653_57fc7b8e5d484c909b615d8633c01d51.html

aa <- allFit(collnearm1$full_model)

is.OK <- sapply(aa,is,"merMod")

aa.OK <- aa[is.OK]

lapply(aa.OK,function(x) x@optinfo$conv$lme4$messages)

# several optimizers led to the convergence of the model (NULL)

# we try re-running the model with the following optimizer:nlminbwrap

collnearm2 <- mixed(collnear ~ velocityc + horizangleabsc + time_decc + (1|species), data = binarydata, method = "LRT", family = binomial, control = glmerControl(optimizer = "nlminbwrap"), expand_re = TRUE)

anova(collnearm2)

# no issues of convergence with the nlminbwrap optimizer

##### we try different optimizers following this approach:

## https://joshua-nugent.github.io/allFit/

aa <- allFit(collnearm1$full_model)

is.OK <- sapply(aa,is,"merMod")

aa.OK <- aa[is.OK]

lapply(aa.OK,function(x) x@optinfo$conv$lme4$messages)

diff_optims <- allFit(collnearm1$full_model, maxfun = 1e5)

diff_optims_OK <- diff_optims[sapply(diff_optims, is, "merMod")]

lapply(diff_optims_OK, function(x) x@optinfo$conv$lme4$messages)

convergence_results <- lapply(diff_optims_OK, function(x) x@optinfo$conv$lme4$messages)

working_indices <- sapply(convergence_results, is.null)

if(sum(working_indices) == 0){

print("No algorithms from allFit converged. You may still be able to use the results, but proceed with extreme caution.")

first_fit <- NULL

} else {

first_fit <- diff_optims[working_indices][[1]]

}

first_fit

# the parameter estimates are the same as obtained following the first approach:

summary (collnearm2$full_model)

# attributes of model object

attributes (collnearm2$full_model)

#### parameters in odds ratio scales for effect size reporting

tidy(collnearm2$full_model,conf.int=TRUE,exponentiate=TRUE,effects="fixed")

# Figures

# plotting velocity effects

ggplot(binarydata, aes(x = velocity, y = collnear)) + geom_point() +

stat_smooth(method="glm", method.args=list(family="binomial"), se = TRUE) +

labs(y = "Probability of collision", x = "Velocity (m/s)") + theme_pubr(16) +

scale_x_continuous(breaks=seq(2,12,1))

# plotting angle effects

ggplot(binarydata, aes(x = horizangleabs, y = collnear)) + geom_point() +

stat_smooth(method="glm", method.args=list(family="binomial"), se = TRUE) +

labs(y = "Probability of collision", x = "Horizontal angle of approach (°)") + theme_pubr(16) +

scale_x_continuous(breaks=seq(0,50,5))

# plotting time

ggplot(binarydata, aes(x = time_dec, y = collnear)) + geom_point() +

stat_smooth(method="glm", method.args=list(family="binomial"), se = TRUE) +

labs(y = "Probability of collision", x = "Time of the day") + theme_pubr(16) +

scale_x_continuous(breaks=seq(2,22,2))

###### Dependent variable: probability of visible injury (stunned, died) after birds collided with window

collafterm1 <- mixed(collafter ~ velocityc + horizangleabsc + time_decc + (1|species), data = binarydata, method = "LRT", family = binomial, expand_re = TRUE)

anova(collafterm1)

# we confirm the singularity issue

tt <- getME(collafterm1$full_model,"theta")

ll <- getME(collafterm1$full_model,"lower")

min(tt[ll==0])

# singularity confirmed being zero

# we increase the number of iterations of the model

ss <- getME(collafterm1$full_model,c("theta","fixef"))

m2 <- update(collafterm1$full_model,start=ss,control=glmerControl(optCtrl=list(maxfun=2e4)))

# singularity persists

##### we try different optimizers following this approach:

## https://rstudio-pubs-static.s3.amazonaws.com/33653_57fc7b8e5d484c909b615d8633c01d51.html

aa <- allFit(collafterm1$full_model)

is.OK <- sapply(aa,is,"merMod")

aa.OK <- aa[is.OK]

lapply(aa.OK,function(x) x@optinfo$conv$lme4$messages)

# singularity persists

# singularity should not be caused by collinearity between the independent factors

# as they are not correlated (see above)

# we subset the data to only species with more than one entry,

# which might improve the estimation of the random variance

binarydata1 <- binarydata [which(binarydata$species == "Blue jay" | binarydata$species == "Cardinal" | binarydata$species == "Downy woodpecker" | binarydata$species == "Grackle" | binarydata$species == "Grosbeak"),]

#### we run the mode with more than 1 entry per species

collaftermsubset <- mixed(collafter ~ velocityc + horizangleabsc + time_decc + (1|species), data = binarydata1, method = "LRT", family = binomial, expand_re = TRUE)

anova(collaftermsubset)

# singularity persists

### we run the original model (with all species) with individual factors

collafterm_v <- mixed(collafter ~ velocityc + (1|species), data = binarydata, method = "LRT", family = binomial, expand_re = TRUE)

anova(collafterm_v)

collafterm_a <- mixed(collafter ~ horizangleabsc + (1|species), data = binarydata, method = "LRT", family = binomial, expand_re = TRUE)

anova(collafterm_a)

collafterm_t <- mixed(collafter ~ time_decc + (1|species), data = binarydata, method = "LRT", family = binomial, expand_re = TRUE)

anova(collafterm_t)

# the singularity went away from one of the single-independent factor models

# most importantly, we confirmed that both velociy and horizangleabs yielded the same significant results

# in the single-independent factor versions of the models.

summary (collafterm1)

#### parameters in odds ratio scales for effect size reporting

tidy(collafterm1$full_model,conf.int=TRUE,exponentiate=TRUE,effects="fixed")

# Figures

# plotting velocity effects

ggplot(binarydata, aes(x = velocity, y = collafter)) + geom_point() +

stat_smooth(method="glm", method.args=list(family="binomial"), se = TRUE) +

labs(y = "Probability of visible injury", x = "Velocity (m/s)") + theme_pubr(16) +

scale_x_continuous(breaks=seq(2,12,1))

# plotting angle effects

ggplot(binarydata, aes(x = horizangleabs, y = collafter)) + geom_point() +

stat_smooth(method="glm", method.args=list(family="binomial"), se = TRUE) +

labs(y = "Probability of visible injury", x = "Horizontal angle of approach (°)") + theme_pubr(16) +

scale_x_continuous(breaks=seq(0,50,5))

# plotting time

ggplot(binarydata, aes(x = time_dec, y = collafter)) + geom_point() +

stat_smooth(method="glm", method.args=list(family="binomial"), se = TRUE) +

labs(y = "Probability of visible injury", x = "Time of the day") + theme_pubr(16) +

scale_x_continuous(breaks=seq(2,22,2))
