# Supplementary material for: Opening the black box of bird-window collisions: passive video recordings in a residential backyard
Source: PeerJ. 2022 Dec 20;10:e14604. doi: 10.7717/peerj.14604 (PMC9784330; doi:10.7717/peerj.14604)
Supplement: Supplemental Information 6 [file peerj-10-14604-s006.docx]

Supplementary Material – Metadata from Generalized Linear Models

Dependent variables

– probability of collision, expressed on a scale of 0-1

– probability of visible injury, expressed on a scale of 0-1

Independent variables

– flight velocity as determined from scoring video recordings

horizangle – horizontal angle of approach

– time of the day when the event was recorded

– species of bird observed in the recording
