# Supplementary material for: Opening the black box of bird-window collisions: passive video recordings in a residential backyard
Source: PeerJ. 2022 Dec 20;10:e14604. doi: 10.7717/peerj.14604 (PMC9784330; doi:10.7717/peerj.14604)
Supplement: Supplemental Information 7 [file peerj-10-14604-s007.docx]

SUPPLEMENTAL MATERIAL

**Camera technology and considerations for future bird collision monitoring research**

To capture footage of birds’ behaviour in flight from upper floors of buildings, cameras with pixel-based object motion detection may be more effective than infrared motion detection if placed inside the glass (e.g., Sundaresan, 2020). Infrared motion detection may still be useful if additional cameras can be mounted on the exterior of the structure and cameras can be triggered synchronously. In selecting cameras that will remained armed or in recording mode continuously over a prolonged study period, it is important to balance image quality and frame rate with practical considerations such as available storage and network bandwidth for uploads. It may also be beneficial to include redundant monitoring of the study area by a human observer in case of camera trigger failure.

New community-science tool for improving understanding of how collisions occur in the real world. Large-scale systematic monitoring of real-world locations using video recording equipment may benefit from automated approaches to coding and analysing contents of footage, such as using motion tracking or pose estimation software. Video processing effort can be greatly reduced by selecting sites that minimize the extent of noise and risk of false-positive triggers (e.g., areas with low pedestrian traffic, falling leaves).

Multiple types of motion detection technology may be useful for recording collision events under different scenarios. The present study used home security cameras with built-in motion detection using front-facing infrared sensors. We previously piloted our study at a different location by recording video of windows on a high-rise building from cameras located inside a room behind the glass. We found that the motion detection function in our cameras, which uses infrared, was not sensitive to birds approaching on the opposite side of a multi-pane window. In this scenario, the amplitude of sound produced by collisions on the relatively thick window glass was likely insufficient to trigger audio recording. We therefore switched to recording at a residential setting with cameras positioned outside in order to not be impeded by glass placed between the lenses and the trigger events. However, even with the cameras positioned outside and with audio and motion detection sensitivity adjusted to capture events and filter out background noise, it is likely that some instances of birds interacting with the windows escaped detection. Given that most of the collision and near-miss events that produced triggers and were successfully recorded involved medium to large passerines (i.e., with body mass exceeding 40 grams), it is possible that events involving smaller birds may be less likely to be picked up by the cameras. To limit detection bias, future studies using cameras for monitoring bird collisions should conduct preliminary trials to calibrate the camera trigger sensitivity.

**REFERENCES**

Sundaresan R. 2020. Pixel-based object motion detection and tracking with a moving camera (Unpublished master’s thesis).
